# Supplementary material for: Prolyl Oligopeptidase Regulates Dopamine Transporter Oligomerization and Phosphorylation in a PKC- and ERK-Independent Manner
Source: Int J Mol Sci. 2021 Feb 10;22(4):1777. doi: 10.3390/ijms22041777 (PMC7916783; doi:10.3390/ijms22041777)
Supplement: Supplementary file 1 [file ijms-22-01777-s001.pdf]

Prolyl oligopeptidase regulates dopamine transporter oligomerization and phosphorylation in a PKC and ERK independent manner

Supplementary Material

Ulrika H. Julku[1], Maria Jäntti[1], Reinis Svarcbahs[1], Timo T. Myöhänen[1,2]

Address: [1]Division of Pharmacology and Pharmacotherapy / Drug Research Program
Faculty of Pharmacy
University of Helsinki
Viikinkaari 5E (PO Box 56)
00014 University of Helsinki
FINLAND

[2]Integrative Physiology and Pharmacology Unit / Institute of Biomedicine
Faculty of Medicine
University of Turku
Kiinanmyllyntie 10
FI-20014 University of Turku
FINLAND

Tel: +358 50 4480769

E-mail: timo.myohanen@helsinki.fi

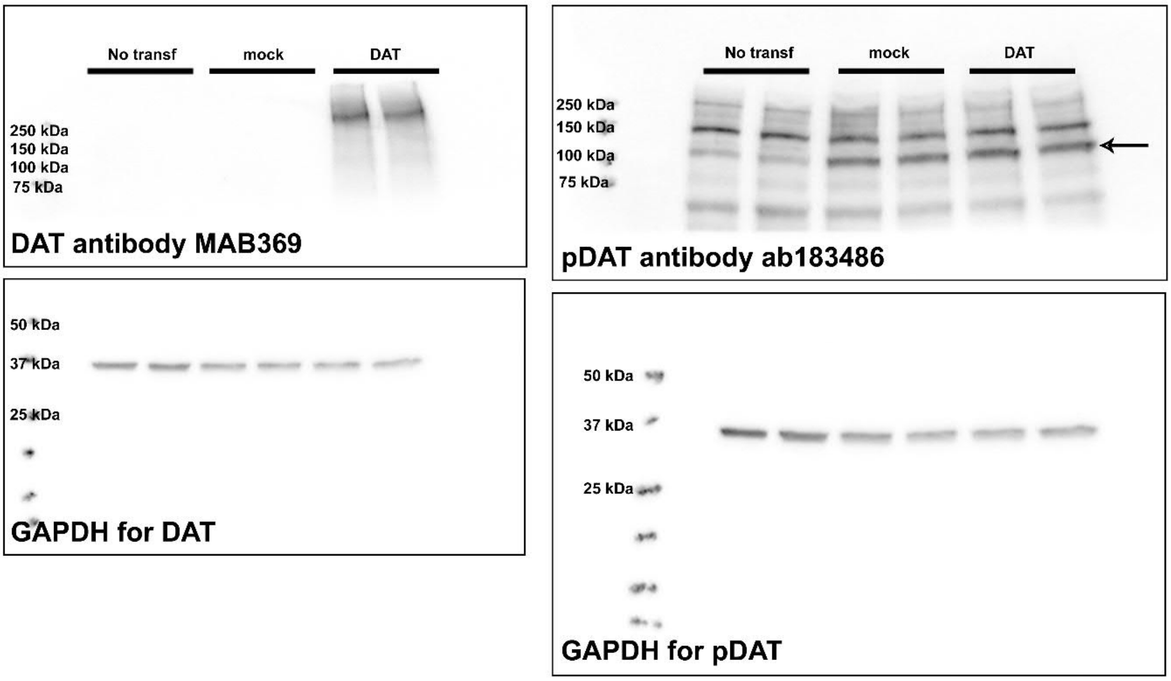

Figure S1. Characterization of DAT (MAB369, Merck Millipore) and pDAT (ab183486, Abcam) antibodies by Western blotting. HEK-293 cells were transfected with mock or DAT plasmid, and also non-transfected cells were used as control. GAPDH was used as a loading control, because expression of β-actin was not similar in transfected and non-transfected cells (data not shown). DAT antibody showed specific binding on DAT transfected cells, but only oligomeric DAT was detected due to high molecular weight band on Western blotting. pDAT antibody had also unspecific binding on Western blotting, but PKC activation induced increase (Fig. 3) confirmed that antibody binds also to pDAT. Unspecific binding of pDAT antibody might prevent detection of small changes in pDAT expression, but massive increase in pDAT expression is still detectable with this antibody.

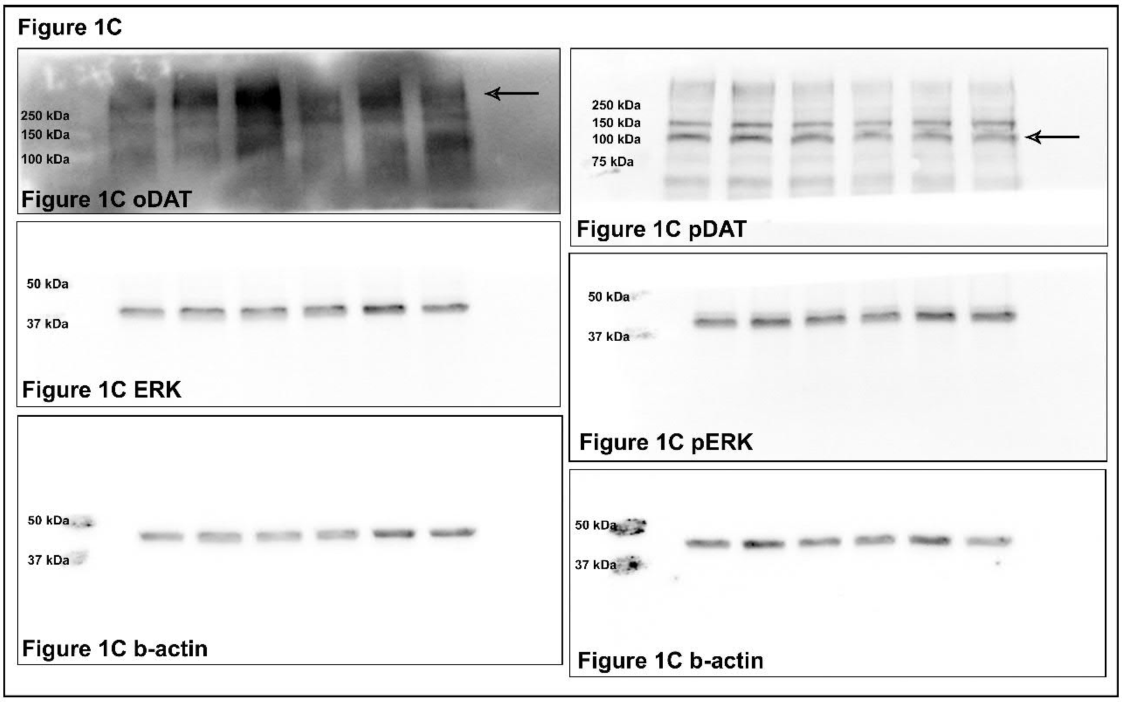

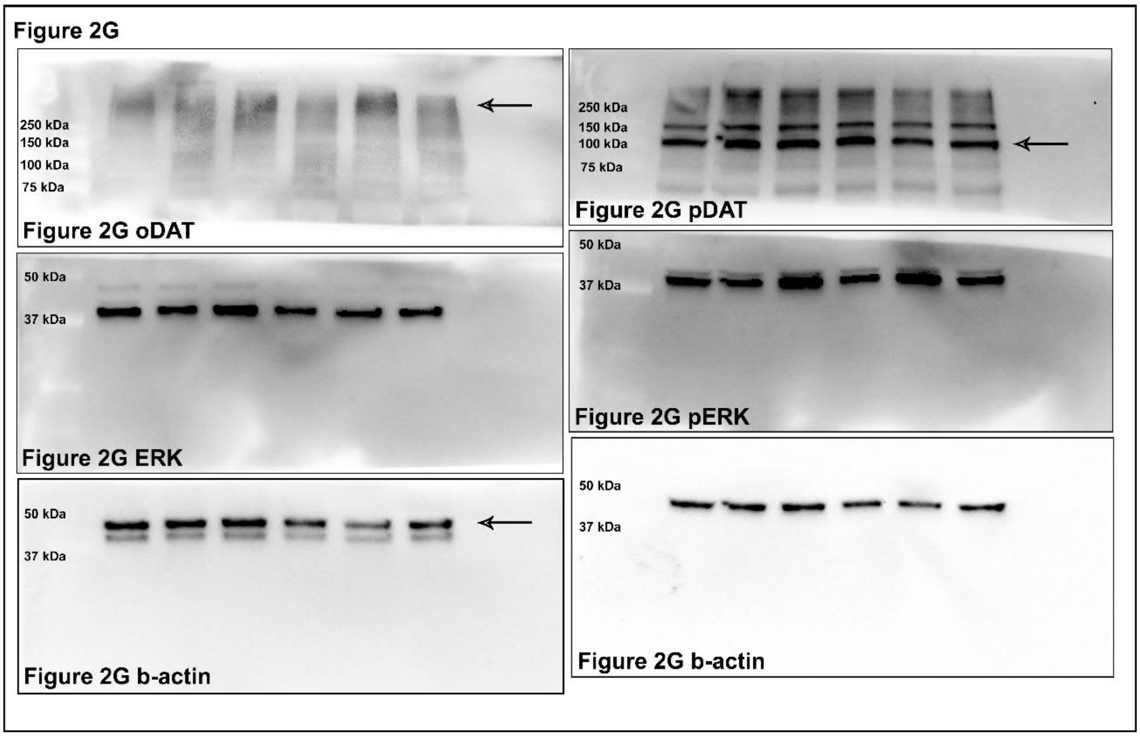

Figure S2. Raw Western blot images for figures 1C and 2G.

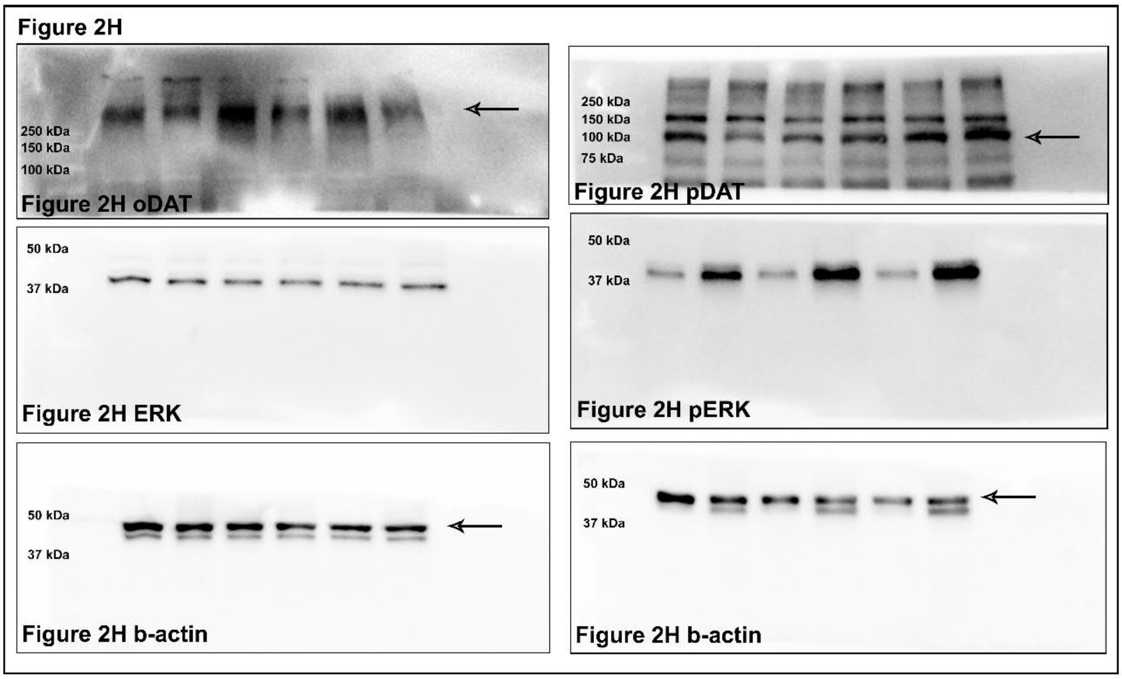

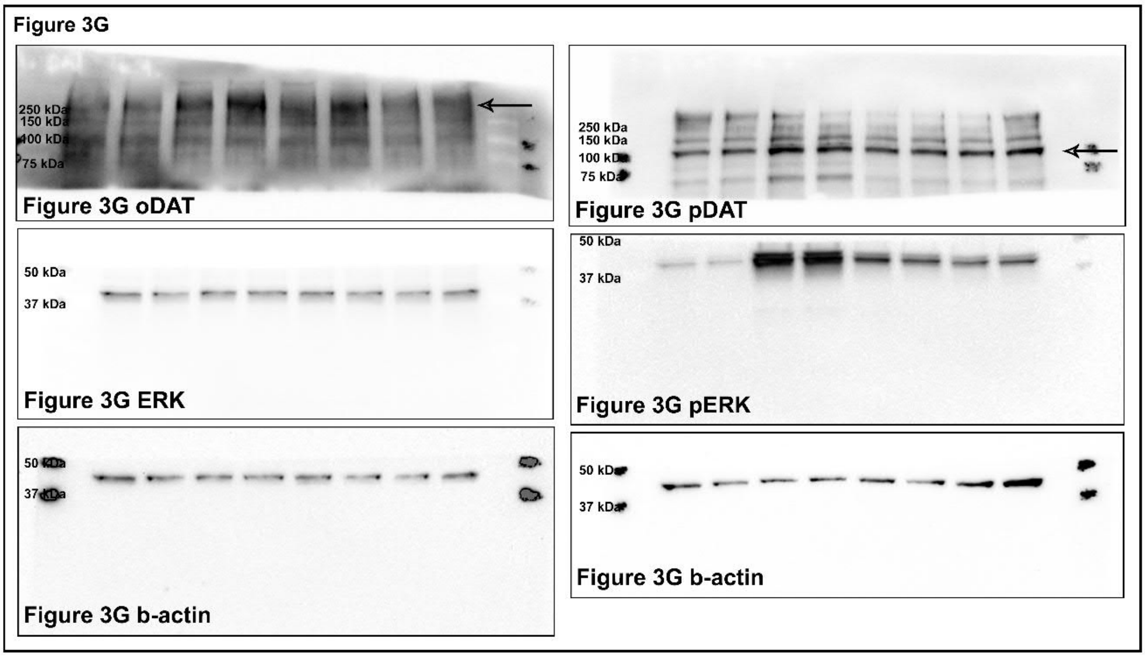

Figure S3. Raw Western blot images for figures 2H and 3G.

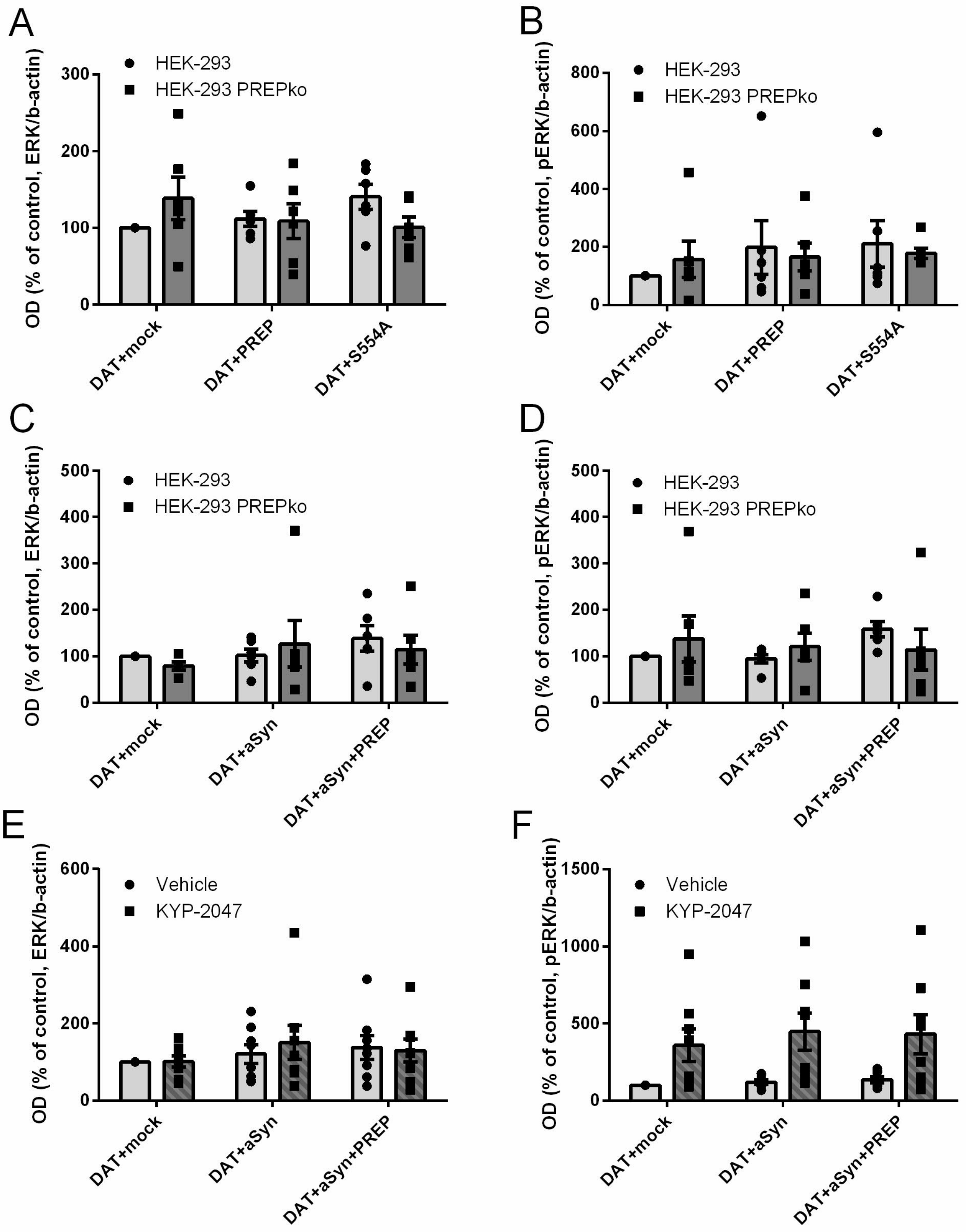

Figure S4. ERK, and pERK were measured by Western blotting in wild-type HEK-293 and stable PREPko cells transfected with DAT and mock, DAT and PREP, or DAT and inactive PREP (S554A-PREP) (A-B), or DAT and mock, DAT and aSyn or DAT, aSyn and PREP (C-D). DAT and mock, DAT and aSyn, and DAT, aSyn and PREP transfected wild-type HEK-

293cells were treated with PREP inhibitor KYP-2047 or vehicle (E-F). Optical density values were normalized to the loading control (β-actin) optical density. Bars represent mean ± SEM, n = 6

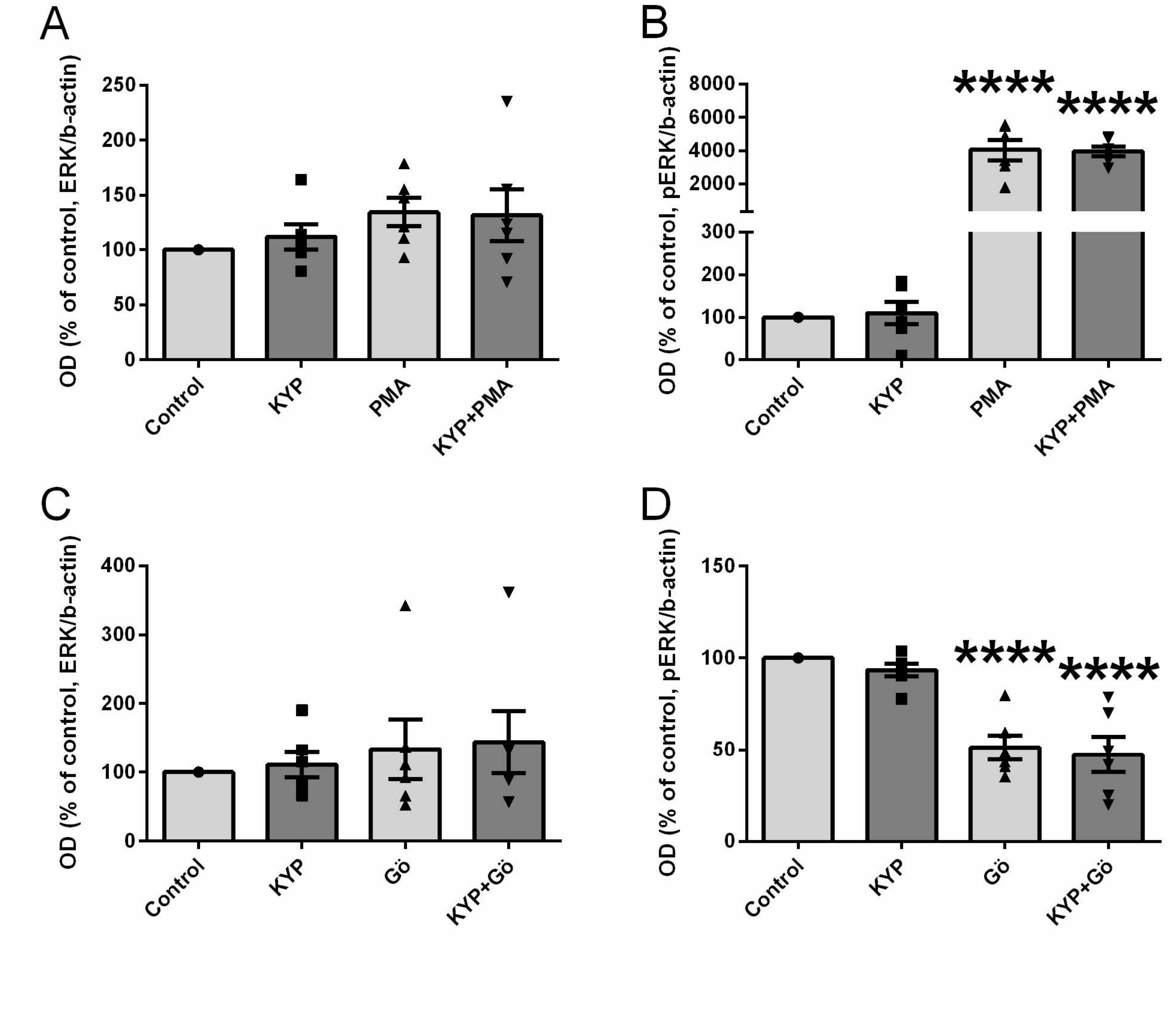

Figure S5. ERK, and pERK were measured by Western blotting in wild-type HEK-293 cells transfected with DAT. Cells were starved in serum-free media for 2 hours before 30 minutes treatment with 1 µM KYP, 1 µM PMA or 1 µM KYP + 1 µM PMA or treated for 30 minutes with 1 µM KYP, 1 µM Gö-9683 or 1 µM KYP + 1 µM Gö-6983 without prior starving. Optical density values were normalized to the loading control (β-actin) optical density. KYP or PMA did not have an effect on ERK A) but PMA and combination of KYP and PMA increased pERK (B). KYP or Gö-6983 did not have an effect on ERK (C) but Gö-6983 and combination of KYP and Gö-6938 decreased phosphorylated ERK (D). Bars represent mean ± SEM, n = 6, ****p < 0.0001, one-way ANOVA, Tukey´s post-hoc test.
